# Changes in Morphology, Metabolism and Composition of Cuticular Wax in Zucchini Fruit During Postharvest Cold Storage

**DOI:** 10.3389/fpls.2021.778745

**Published:** 2021-12-07

**Authors:** Fátima Carvajal, Alejandro Castro-Cegrí, Raquel Jiménez-Muñoz, Manuel Jamilena, Dolores Garrido, Francisco Palma

**Affiliations:** ^1^Department of Plant Physiology, Facultad de Ciencias, University of Granada, Granada, Spain; ^2^Department of Biology and Geology, Agrifood Campus of International Excellence (CeiA3), University of Almería, Almería, Spain

**Keywords:** *Cucurbita pepo*, postharvest, cold stress, cuticle, cuticular wax, alkanes

## Abstract

Cuticle composition is an important economic trait in agriculture, as it is the first protective barrier of the plant against environmental conditions. The main goal of this work was to study the role of the cuticular wax in maintaining the postharvest quality of zucchini fruit, by comparing two commercial varieties with contrasting behavior against low temperatures; the cold-tolerant variety ‘Natura’, and the cold-sensitive ‘Sinatra’, as well as ‘Sinatra’ fruit with induced-chilling tolerance through a preconditioning treatment (15°C for 48 h). The freshly-harvested ‘Natura’ fruit had a well-detectable cuticle with a significant lower permeability and a subset of 15 up-regulated cuticle-related genes. SEM showed that zucchini epicuticular waxes mainly consisted of round-shaped crystals and clusters of them, and areas with more dense crystal deposition were found in fruit of ‘Natura’ and of preconditioned ‘Sinatra’. The cuticular wax load per surface was higher in ‘Natura’ than in ‘Sinatra’ fruit at harvest and after 14 days at 4°C. In addition, total cuticular wax load only increased in ‘Natura’ and preconditioned ‘Sinatra’ fruit with cold storage. With respect to the chemical composition of the waxes, the most abundant components were alkanes, in both ‘Natura’ and ‘Sinatra’, with similar values at harvest. The total alkane content only increased in ‘Natura’ fruit and in the preconditioned ‘Sinatra’ fruit after cold storage, whereas the amount of total acids decreased, with the lowest values observed in the fruit that showed less chilling injury (CI) and weight loss. Two esters were detected, and their content also decreased with the storage in both varieties, with a greater reduction observed in the cold-tolerant variety in response to low temperature. Gene expression analysis showed significant differences between varieties, especially in *CpCER1-like* and *CpCER3-like* genes, involved in alkane production, as well as in the transcription factors *CpWIN1-like* and *CpFUL1-like*, associated with cuticle development and epidermal wax accumulation in other species. These results suggest an important role of the alkane biosynthetic pathway and cuticle morphology in maintaining the postharvest quality of zucchini fruit during the storage at low temperatures.

## Introduction

The cuticle is a barrier that was first developed in plants during their colonization to dry land. Due to its lipidic composition, the cuticle not only prevents dehydration but it is also a protective barrier against biotic and abiotic stress, and plays a role in preventing damage from mechanical stress (Domínguez et al., 2011). The cuticle is synthesized by the epidermal cell layer and is composed of a cutin matrix, in contact with the cell wall, and a complex group of different cuticular waxes that embed and cover the cutin matrix, constituting intracuticular and epicuticular waxes, respectively, (Ensikat et al., 2006; Koch and Ensikat, 2008; Buschhaus and Jetter, 2011). Cuticular waxes consist of a mixture of very-long chain fatty acids (VLCFAs) and its derivatives, which are synthesized by two pathways: the alcohol-forming pathway, which generates primary alcohols and esters, and the alkane-forming pathway, which produces aldehydes, alkanes, secondary alcohols, and ketones (Yeats and Rose, 2013). The biosynthesis of cuticular wax by one metabolic pathway or another may be regulated by different compounds, such as abscisic acid (Wang et al., 2015a; Romero and Lafuente, 2020). The predominant components of the waxes in the cuticle of many fruits are VLC-alkanes, which account for 50–80% of total wax content, and nonacosane (C29) or hentriacontane (C31), which are the majority. In addition, fatty acid alcohols, aldehydes, ketones, and triterpenoids have also been detected (Bauer et al., 2004; Parsons et al., 2012; Wang et al., 2015a; Wu et al., 2018).

In fleshy fruits, cuticular waxes play a crucial role in minimizing water loss/uptake through the surface, providing mechanical support, preventing fruit softening, and increasing pathogen resistance (Martin and Rose, 2013; Tafolla-Arellano et al., 2017; Trivedi et al., 2019). In blueberry fruits, wax removal decreased postharvest fruit quality during cold storage, accelerating water loss and decay (Chu et al., 2018). The cuticle in fruits is usually thicker than in leaves, and the structure and composition of cuticular wax has been shown to be closely related to the postharvest quality of fruit (Lara Ayala et al., 2014, 2019). The composition of cuticular wax varies widely among fruit species and cultivars (Trivedi et al., 2019), and many of the cuticular properties are affected by wax composition. The presence of long-chain alkanes and aldehydes has been found to increase water impermeability of fruit cuticles. With regard to this, a positive correlation between cuticle composition and water loss has been reported, finding that the alkane content was a significant determinant of water permeability in pepper (Parsons et al., 2012).

With respect to the biosynthetic pathway of the cuticular wax, most of the studies have been conducted in *Arabidopsis thaliana*, and many of the genes involved in alkane biosynthesis have been identified from *eceriferum* mutants (McNevin et al., 1993). Among them, CER2, CER26, CER1, and CER3 have been found to be determinant genes for the proper development of the cuticle, and shown to be involved in alkane biosynthesis. CER2 mutants were shown to have a severe wax deficiency and accumulated 26C wax components, being deficient for components longer than 28C (Haslam et al., 2017). CER26 mutants were shown to have a similar behavior to CER2, being specifically affected in the VLC-fatty acid elongation process (Pascal et al., 2013). Mutants with a CER1 loss-of-function have cuticles with a reduced number of alkanes and its derivatives (Bourdenx et al., 2011). CER3/WAX2 is also involved in the production of wax, with the *wax2* mutants having a decreased wax content of about 80% compared to wild type plants (Chen et al., 2003). WAX2 catalyzes the reduction of VLC-acyl-CoAs to intermediate VLC-aldehydes, which are then converted to alkanes through decarbonylation by CER1 (Bernard et al., 2012). The most important genes belonging to the alkane-forming pathway that lead to the formation of VLC-alkanes are CER1 and CER3. They interact physically, and it has been proposed that they are the core components of a VLC-alkane synthesis complex (Bernard et al., 2012). CER3 catalyzes the reduction of VLC-acyl-CoAs, forming aldehydes that are decarbonylated by CER1 to VLC-alkanes (Bernard et al., 2012). Homologues to these genes have been characterized in other species; in cucumber, the importance of CER1 and CER3 in wax biosynthesis and its implication in stress and water permeability has been investigated. The *CsCER1* transcript was induced by abiotic stresses, and transgenic RNAi lines with a knock down of the transcript were altered in cuticular wax biosynthesis (Wang et al., 2015a). *CsWAX2* (CER3) also played a significant role in the plant’s response to biotic and abiotic stresses (Wang et al., 2015b). Along with the genes encoding for enzymes responsible for the biosynthesis of wax components, several transcription factors have been identified as regulators of the cuticular wax biosynthesis (Hen-Avivi et al., 2014), such as members of the SHINE clade of AP2-domain transcription factors, which are responsible for cuticle formation and deposition in several fruits, such as tomato, sweet cherry, apple, and mango (Alkio et al., 2012; Shi et al., 2013; Lashbrooke et al., 2015; Tafolla-Arellano et al., 2017).

During the postharvest cold storage of *Cucurbita pepo*, the fruits experience chilling injury (CI), a disease triggered by low temperatures, which affects fruit quality and is responsible for economic losses, with the intensity of the damages dependent on the variety (Carvajal et al., 2011). The search for resistance and improvement of zucchini fruit quality during postharvest cold storage has resulted in the selection of varieties that are more resistant to CI, such as ‘Natura’, and this resistant variety has been compared to a ‘Sinatra’, a very sensitive variety. The biochemical and genetic differences that make these two varieties resistant and sensitive, respectively, have been thoroughly investigated (Palma et al., 2014a,b; Carvajal et al., 2017), and different treatments have been applied to search for improvements in the quality of the fruit during cold storage. Fruit from the cold sensitive ‘Sinatra’, when preconditioned at 15°C for 48 h before storage at 4°C (PCT) were able to resist cold storage and improved in quality during the postharvest period (Carvajal et al., 2015). In a transcriptomic study, several genes that were differentially expressed during cold storage were selected (Carvajal et al., 2018b), and among them genes related to cuticular wax deposition were found. Since one of the differences between ‘Natura’ and ‘Sinatra’ was the lower weight loss in the resistant ‘Natura’, one of the possibilities for cold resistance was a difference in the cuticle between these varieties. Based on this hypothesis, in a first approach, we detected that long-term cold storage diminished the expression of genes belonging to the fatty acid elongation complex and the ECERIFERUM proteins 1 and 3 (CER1 and CER3; Carvajal et al., 2018a). To shed light on the involvement of the cuticle in the maintenance of postharvest quality of zucchini fruit, the aim of the present work was to analyze the cuticle properties and the differences in cuticular waxes between the varieties ‘Natura’ and ‘Sinatra’, as well as in PCT ‘Sinatra’ fruit. Changes in permeability, structure, composition, and expression of cuticle-related genes will be discussed in terms of cold resistance.

## Materials and Methods

### Fruit Material

Zucchini fruit (*Cucurbita pepo* L. Morphotype *Zucchini*) from the commercial varieties ‘Natura’ (EnzaZaden) and ‘Sinatra’ (Clause-Tezier) were provided by “Hortofrutícola La Ñeca S.L.” Freshly-harvested fruits, free of disease symptoms, mechanical damage and with uniform length, were randomly divided into replicates and stored in a temperature-controlled chamber in permanent darkness at 4°C and 85–90% RH for 14d. In addition to the control fruit in the case of the ‘Sinatra’ variety, a group of fruits were also preconditioned at 15°C for 48 h before storage at 4°C (PCT), according to Carvajal et al. (2015). For microscopy and chlorophyll leaching assays, 10 fruits were analyzed per storage time, variety, and treatment, and the experiment was conducted twice. Two more experiments using 18 fruits (three replicates of six fruits each) per storage time, variety, and treatment were performed to analyze the cuticular wax load and composition and to sample exocarp tissue for RNA extraction. For the latter, the tissue was frozen in liquid N_2_, ground, and stored at −80°C.

### Chlorophyll Leaching Assay

Freshly-harvested ‘Natura’ and ‘Sinatra’ fruit were used to assess epidermal permeability through the chlorophyll efflux assay, according to Kosma et al. (2009) with some modifications. Three cylinders were taken from the proximal, equatorial, and distal zones of each fruit using a cork borer. Both cylinder sides were excised with a razor blade resulting in two 3 mm thick discs per cylinder. Discs were washed 3 times in distilled water for 5 min and, lastly, immersed in equal volumes of 80% ethanol and maintained at room temperature in the dark with a gentle shake. The absorbance at 647 and 664 nm of the solution was measured at 15, 30, 60, 120, 180 min, and after 24 h. The micromolar concentration of total chlorophyll was calculated by the equation: total chlorophyll = 7.93(A_664_) + 19.53(A_647_) (Lolle et al., 1997). The amount of chlorophyll leached was expressed as a percentage of the total chlorophyll extracted after 24 h.

### Microscopy

For light microscopy, exocarp tissue was fixed, embedded, and cryosectioned according to Buda et al. (2009). Briefly, 2–3 mm exocarp cubes were sliced with a scalpel from the equatorial region of ten fruits per condition. The cubes were transferred to FAA fixative (5% formaldehyde, 5% acetic acid, and 45% ethanol) and vacuum infiltrated for 20 min. The FAA was replaced with fresh fixative and the samples were kept overnight at 4°C. After this, a sucrose gradient (10 and 20% in PBS buffer) was used following the same conditions than for the fixation process. FSC 22 Frozen Section medium (Leica) was used to fill the cryo-molds. Cryosections (40 and 50 μm) were obtained using a Leica CM1950 cryostat, transferred to Superfrost^®^ Plus (Thermo Fisher Scientific, MA, United States), and stained with a 0.2% (w/v) Oil Red solution in 60% (v/v) isopropanol (Fukumoto and Fujimoto, 2002). The stained slides were visualized using an Axioscope 5 microscope and captured with an Axiocamp 305 color camera.

For scanning electron microscopy (SEM), exocarp tissue was fixed in 2.5% (w/v) glutaraldehyde in 0.1 M cacodylate buffer pH 7. The samples were rinsed four times in cacodylate buffer, dehydrated in a graded ethanol series, and critical point dried in CO_2_ in a Polaron CPD 7501 dryer. Afterwards, they were mounted onto steel plates, evaporated with charcoal in a Hitachi evaporator, and sputter-coated with gold palladium in a Polaron Unit SEM Coating E5000. The samples were visualized and recorded using a Zeiss SUPRA40VP scanning electron microscope.

### Cuticular Wax Analysis

Before the extraction, the fruits were washed in distilled water for 5s and air dried. Then, epicuticular and intracuticular compounds (cuticular wax) were extracted by organic extraction. The cuticular wax was extracted by dipping the fruit for 1 min at room temperature in a chloroform:methanol mixture (2:1, v/v) containing 60 μg of tetracosane, lignoceryl alcohol, and lignoceric acid as internal standards. After the extraction, the exocarp of each fruit was separated using a vegetable peeler and photographed to measured total fruit surface using the ImageJ software (Schneider et al., 2012). The extraction solution was evaporated under a stream of nitrogen, resuspended in chloroform:distilled water (2:1, v/v), and the chloroform phase separated (Yeats et al., 2010). The solution containing the waxes was evaporated under a stream of nitrogen, and after that, maintained under vacuum conditions at room temperature. The wax load was determined gravimetrically.

Cuticular wax (1 mg) was derivatized with N,O-Bis(trimethylsilyl)trifluoroacetamide:pyridine (1,2) for 40 min at 70°C. After evaporating to dryness under nitrogen flow, the samples were re-dissolved in chloroform and injected into a gas chromatograph (Bruker 456-GC), coupled with an EVOQ triple quadrupole (TQ) detector and a ZB-5MS capillary column (30 m × 0.250 mm i.d. × 0.25 μm). Helium was used as a carrier gas at a flow rate of 1.2 mlmin^−1^. The running program was set as follows: the temperature was set to 50°C for 2 min and then a ramping period was executed from the initial 50°C–200°C at a rate of 10°C min^−1^, and finally increased to 320°C at a rate of 8°C min^−1^, and held at this temperature for 20 min. The following parameters were employed: inlet, MS transfer line, ion source, quadrupole temperatures were 260°C, 260°C, 250°C, and 42°C, respectively. Electron impact (EI) ionization voltage was 70eV, and the m/z range was set between 50 and 500.The identification of the main wax components was performed by comparing their mass spectra with the National Institute of Standards and Technology Version 2.3 library database, or from previously published data. Quantitative determination was achieved by comparing the peaks with the known value of an internal standard.

### RNA Extraction and Gene Expression Analysis

Total RNA from the exocarp of each biological sample was extracted, treated with RNAse-Free DNAse, and purified using TRIsure^™^ reagent (Bioline) and the Direct-zol^™^ RNAMiniprep kit (Zymo Research). The quality and quantity of RNA was determined by agarose gel electrophoresis and a NanoDrop Lite spectrophotometer (Thermo Fisher Scientific). First-strand cDNA was synthesized from 1 μg total RNA using PrimeScript^™^ RT Master Mix (Takara). Primer pairs for the VLC-alkane biosynthesis genes *CpCER2-like* (*Cp4.1LG02g03940*), *CpCER26-like* (*Cp4.1LG17g02960*), *CpCER1-like* (*Cp4.1LG17g02820*), and *CpCER3-like* (*Cp4.1LG04g12660*), and the cuticle-related transcription factors *CpSHINE2* (*Cp4.1LG06g03420*), *CpWIN1-like* (*Cp4.1LG04g09380*), and *CpFUL1-like* (*Cp4.1LG03g15390*) were designed using the Primer3 web tool^1^ and are listed in Supplementary Table S1. For qRT-PCR, the amplifications were run in an iCycler iQ thermal cycler (Bio-Rad). Quantification was performed with the iCycler iQTM associated software (Real Time Detection System Software, version 2.0). The relative gene expression was calculated using non-stored ‘Natura’ fruit as the calibration sample. *EF-1α* was used as the internal reference gene for normalizing the transcript profiles following the 2^−ΔΔCt^ method (Livak and Schmittgen, 2001).

### Statistical Analysis

The experimental design was completely randomized. The data were subjected to an ANOVA using the SPSS 15.0 software (SPSS Inc.). The means were compared with Duncan’s least significant difference test, and differences at *p* < 0.05 were considered significant.

## Results

### Differences in Zucchini Fruit Cuticle Properties Between Varieties Before Storage

**Figure 1:**
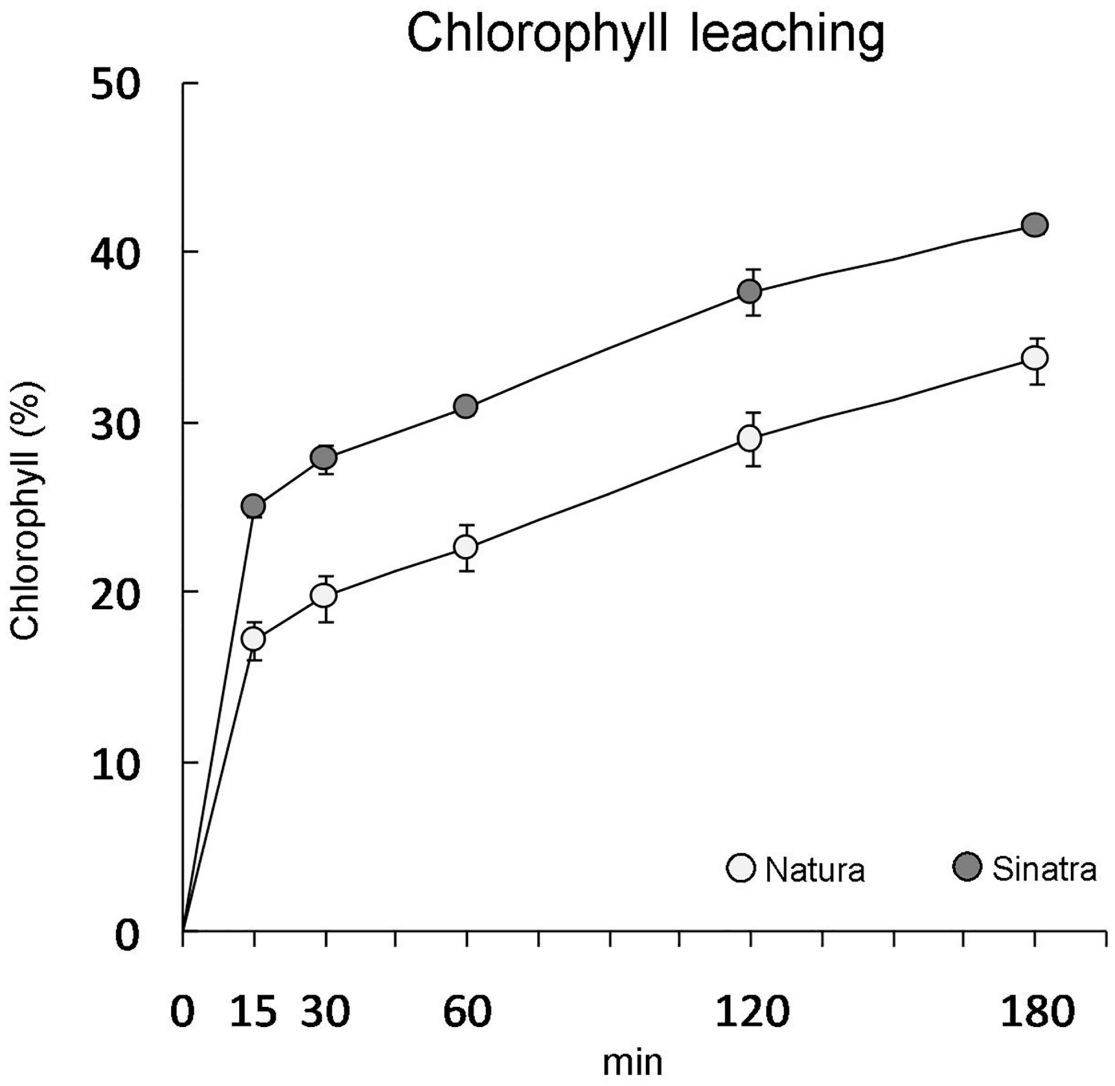
Chlorophyll leaching of ‘Natura’ and ‘Sinatra’ fruit at harvest expressed as a percentage of total chlorophyll extracted after 24 h. Data presented are means ± SE of ten fruits. Differences between varieties were significant for every time assayed according to Duncan’s test (*p* < 0.05).

The outer surface of the epidermal cells was covered by a continuous bright-red structure in all the cryosections observed from the fruit of the cold-resistant variety ‘Natura’, indicating the presence of a well-detectable cuticle (Supplementary Figure S1). On the contrary, few cryosections showed a clearly distinguishable cuticle in fruit from the cold-sensitive variety ‘Sinatra’ (Supplementary Figure S1). Although in both varieties the cuticle was not thick enough to be measured, in ‘Natura’ it seemed thicker than in ‘Sinatra’. The chlorophyll extraction rate was significantly higher in ‘Sinatra’ fruit (Figure 1), supporting the structural observations made with light microscopy.

In a previous study, we conducted a transcriptomic comparison of ‘Natura’ and ‘Sinatra’ exocarp before and after storage by RNA-Seq (Carvajal et al., 2018b). The result showed that the GO term ‘fatty acid biosynthetic process’ was overrepresented when comparing differentially expressed genes (DEGs) that were up-regulated in freshly-harvested ‘Natura’ versus ‘Sinatra’ fruit. In this work, we searched for possible cuticle-related DEGs between varieties at harvest and compared with their respective homologues in *Arabidopsis thaliana* or *Solanum lycopersicum* (Supplementary Table S2). From the 237 DEG annotated that were significantly up-regulated in ‘Natura’ with respect to ‘Sinatra’, a total of 15 were related to cuticle biosynthesis or its regulation, constituting 6.3% of the total. Among them, the transcript corresponding to the fatty acid hydroxylase homolog to the very-long-chain aldehyde decarbonylase CER1 from *Arabidopsis* had the highest differential accumulation. We found four DEGs encoding for 3-ketoacyl-CoA synthases; homologues to 3-ketoacyl-CoA synthase 6, 3-ketoacyl-CoA synthase 10, 3-ketoacyl-CoA synthase 19, and 3-ketoacyl-CoA synthase 20 from *Arabidopsis*; three GDSL esterases/lipases, two of them similar to OSP1; and an O-acyltransferase WSD1. Although most of the DEGs were related to waxes, we also found two cutin-specific genes; a cytochrome P450 and a HXXXD-type acyl-transferase similar to cytochrome P450 86A2, and a BAHD acyltransferase DCR, respectively. Two genes encoding non-specific lipid-transfer proteins were also detected. Regarding the regulation of the cuticle formation, two transcription factors were also differentially expressed; ethylene-responsive WIN1 and MADS box, with a high homology to *Arabidopsis* WIN1 and tomato FRUITFULL-like MADS-box 1. On the other hand, the exocarp of ‘Sinatra’ fruit had 178 DEGs up-regulated with respect to ‘Natura’, of which only one could be associated with cuticle metabolism, constituting 0.56% of the total. This DEG corresponded to a Membrane Bound O-acyl transferase (MBOAT).

### Cuticular Wax Morphology and Composition Before and After Storage at Low Temperature

**Figure 2:**
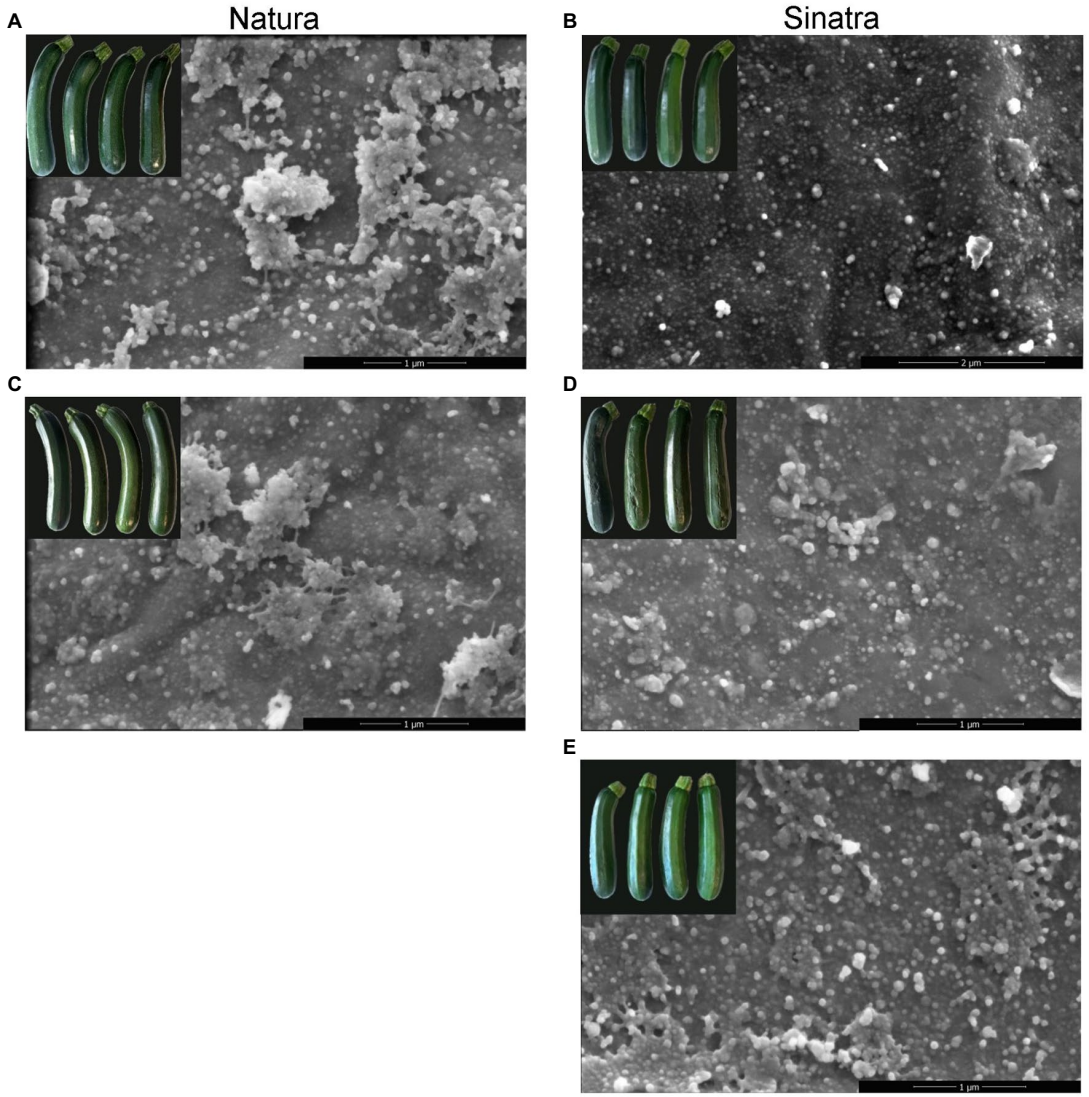
Scanning electron micrographs of zucchini exocarp surface from ‘Natura’ and ‘Sinatra’ fruit, at harvest **(A,B)** and after 14 days of storage at 4°C **(C,D)** including preconditioned ‘Sinatra’ **(E)**. Scar bars: 1 or 2 μm.

SEM observations revealed that epicuticular wax in zucchini fruit mainly consisted of round-shaped or granule-like crystals scattered over the fruit surface (Figure 2). All samples analyzed had areas differing in the abundance of the wax coverage; however, in the ‘Natura’ fruit surface, the areas richer in agglomerations of granule-like crystals were more abundant in both conditions before and after the cold storage (Figures 2A,C). PCT fruit also had more regions whose surface was more enriched with epicuticular crystals (Figure 2D) when compared with non-treated ‘Sinatra’ fruit (Figure 2E). This finding suggests the formation of new waxes onto the cuticle surface what is also supported by their appearance when the images were analyzed under less magnification (Supplementary Figure S2).

**Figure 3:**
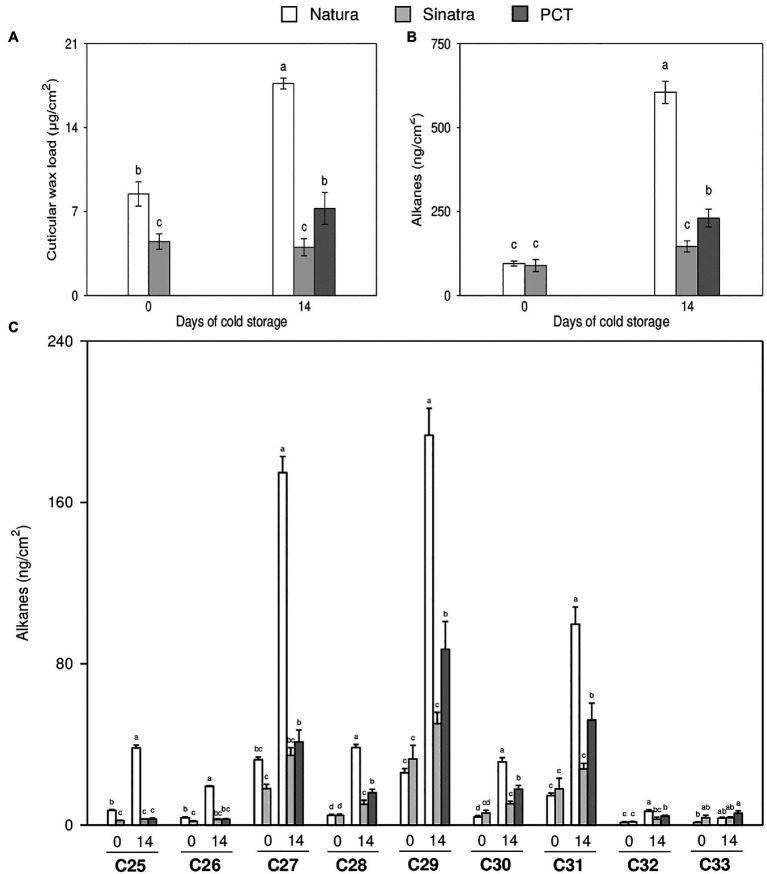
Cuticular wax load **(A)**, total alkane content **(B)**, and individual alkanes per surface **(C)** measured in zucchini ‘Natura’ and ‘Sinatra’ fruit, at harvest and after 14 days of storage at 4°C, including preconditioned ‘Sinatra’. Data presented are means ± SE of triplicate samples of six fruits each. Different letters indicate significant differences according to Duncan’s test (*p* < 0.05).

The total cuticular wax load per surface area of the two zucchini varieties was different (Figure 3A). The cold-tolerant variety ‘Natura’ showed significantly more content of cuticular wax than the cold-sensitive variety ‘Sinatra’ at harvest and after 14 days of exposure to chilling. Total cuticular wax load only increased in ‘Natura’ during cold storage, as shown by values that doubled those exhibited in fruit at harvest after the 14 days of exposure. By contrast, ‘Sinatra’ fruit did not change significantly with storage. After 14 days at 4°C, ‘Natura’ fruit and the PCT exhibited significantly higher cuticular wax content than ‘Sinatra’ fruit, increasing about 4.4- and 1.8-fold, respectively.

Alkanes were one of the most important VLC aliphatic compounds in the cuticular wax of zucchini fruit. The total concentration of alkanes did not differ significantly between the two varieties in fruit at harvest (Figure 3B). The storage at low temperature induced the alkane-forming pathway in ‘Natura’ fruit, reaching very high levels, about 7-fold with respect to freshly-harvested fruit. On the contrary, ‘Sinatra’ fruit showed no significant changes in response to chilling stress, but with the preconditioning treatment, it increased 2.6-fold.

Nine VLC-alkanes ranging from C25 to C33 were detected in zucchini fruit (Figure 3C). Among the different alkanes identified, heptacosane (C27), nonacosane (C29), and hentriacontane (C31) were the most abundant in both varieties. Comparing the freshly-harvested fruit of the two varieties, no significant differences were observed in the amounts of heptacosane, nonacocosane and hentriacontane. However, these alkanes increased 5.5-, 8- and 7-fold respectively, in ‘Natura’ fruit kept at low temperature. The preconditioned fruit showed the same response as that observed in the cold-tolerant variety, but the increase was lower, 2.3-, 2.7 and 3-fold, respectively. By contrast, in the cold-sensitive variety, the content of these alkanes did not show significant differences between freshly-harvested and fruit stored at 4°C. In addition, the amounts of all alkanes per square cm, except for C28, did not significantly differ in ‘Sinatra’ fruit during cold storage with respect to harvest day.

The analysis of the cuticular wax composition in both zucchini varieties revealed that the fatty acids were the second dominant component (Table 1). Fourteen fatty acids ranging from C14 to C32 were detected in zucchini fruit, with hexadecanoic acid (C16:0), octadecanoic acid (C18:0), and hexacosanoic acid (C26:0) being predominant. The percentage of total fatty acids showed a contrary behavior to the obtained for the percentage of alkanes. The highest value was reached in ‘Sinatra’ fruit at harvest, although it did not change significantly during cold storage. The percentage of total fatty acids was lower in ‘Natura’ and PCT than in ‘Sinatra’. In the cold-tolerant variety, a 13% reduction was also detected as a consequence of the storage at low temperature, whereas in PCT, a 17% reduction was observed with respect to freshly-harvested fruit. Studying the profile of saturated fatty acids in the cold-sensitive variety, all saturated fatty acids did not show statistical significant differences between freshly-harvested and fruit stored at 4°C. However, the preconditioning treatment reduced the percentage of hexadecanoic, heptadecanoic, octadecanoic, and octacosanoic acids.

**Table 1:** Relative content (%) of wax compounds in Natura (cold-tolerant variety), Sinatra (cold-sensitive variety) and PCT (preconditioned treatment in Sinatra) zucchini fruit at harvest and stored during 14 days at 4°C.

		At harvest		After 14 days at 4°C			LSD (*p* <0.05)
		Natura	Sinatra	Natura	Sinatra	PCT	
Alkanes		34.0^c^	30.0^c^	70.6^a^	35.7^c^	52.9^b^	08.4
Pentacosane	C25	02.6^b^	00.8^c^	04.5^a^	00.8^c^	00.7^c^	00.6
Hexacosane	C26	01.3^b^	00.6^c^	02.3^a^	00.7^c^	00.7^c^	00.2
Heptacosane	C27	11.7^b^	06.2^d^	20.4^a^	08.4^cd^	09.5^bc^	02.4
Octacosane	C28	01.7^d^	01.6^d^	04.5^a^	02.5^c^	03.7^b^	00.3
Nonacosane	C29	09.3^b^	11.1^b^	22.5^a^	12.3^b^	20.0^a^	03.4
Triacontane	C30	01.4^e^	02.0^d^	03.6^b^	02.6^c^	04.1^a^	00.4
Hentriacontane	C31	05.2^b^	06.0^b^	11.6^a^	06.8^b^	11.9^a^	02.4
Dotriacontane	C32	00.4^c^	00.5^bc^	00.8^ab^	00.7^bc^	01.0^a^	00.2
Tritriacontane	C33	00.4^b^	01.2^a^	00.4^b^	00.9^a^	01.3^a^	00.5
Fatty Acids		29.8^b^	44.5^a^	17.1^c^	38.5^a^	27.5^b^	08.0
Tetradecanoic acid	C14:0	00.4^a^	00.5^a^	00.3^a^	00.2^a^	00.2^a^	00.6
Pentadecanoic acid	C15:0	00.2^a^	00.3^a^	00.2^a^	00.1^a^	00.2^a^	00.4
Hexadecanoic acid	C16:0	09.4^a^	10.7^a^	04.5^b^	07.9^ab^	04.1^b^	04.0
Heptadecanoic acid	C17:0	0nd	00.5^a^	00.1^b^	00.4^a^	00.2^b^	00.1
Octadecanoic acid	C18:0	04.0^b^	07.3^a^	02.6^b^	06.0^a^	03.0^b^	01.3
Docosanoic acid	C22:0	00.6^b^	01.9^a^	00.4^b^	01.7^a^	01.3^a^	01.0
Pentacosanoic acid	C25:0	00.6^b^	01.1^a^	00.5^b^	01.2^a^	01.0^a^	00.4
Hexacosanoic acid	C26:0	04.2^b^	06.9^ab^	03.6^b^	09.1^a^	06.5^ab^	03.5
Heptacosanoic acid	C27:0	00.5^b^	01.1^a^	00.4^b^	01.1^a^	01.1^a^	00.4
Octacosanoic acid	C28:0	01.4^c^	05.6^a^	01.7^c^	04.6^ab^	03.6^b^	01.2
Triacontanoic acid	C30:0	02.6^a^	01.4^bc^	00.7^c^	01.6^bc^	02.0^ab^	00.9
Dotriacontanoic acid	C32:0	00.7^b^	02.9^a^	00.7^b^	02.5^a^	02.7^a^	01.0
Palmitoleic acid	C16:1(9)	03.2^a^	01.4^ab^	00.9^b^	00.6^b^	00.6^b^	02.1
Oleic acid	C18:1(9)	01.0^bc^	02.9^a^	00.5^c^	01.5^b^	01.0^bc^	00.6
Fatty Alcohols		02.7^b^	05.2^a^	03.0^b^	06.1^a^	05.1^a^	01.2
Hexacosanol	C26	00.9^ab^	00.7^b^	00.8^b^	01.1^a^	00.8^b^	00.2
Octacosanol	C28	00.8^b^	01.5^a^	00.9^b^	01.8^a^	01.5^a^	00.4
Triacontanol	C30	01.0^b^	03.0^a^	01.3^b^	03.2^a^	02.8^a^	00.6
Esters		19.9^a^	09.3^b^	06.1^d^	07.3^c^	04.6^e^	01.1
1-monopalmitin		00.6^a^	00.6^a^	00.2^b^	00.3^b^	00.2^b^	00.2
1-monostearin		19.3^a^	08.7^b^	05.9^c^	07.0^c^	04.3^d^	01.3

Relative content (%) was regarded as the percentage of each compound compared to the total content of compounds. Values represent means (nd, non-detectable). Different letters indicate significant differences according to Duncan’s test (*p* < 0.05).

Three primary alcohols were found in the cuticular wax of zucchini fruit (Table 1). The fatty alcohols profile was composed of hexacosanol, octacosanol, and triacontanol, but their percentage was relatively low with respect to the other compounds. The proportion of primary alcohols accounted for about 5% of the total wax found in zucchini fruit. The total percentage of alcohols did not change significantly in ‘Natura’ and ‘Sinatra’ fruit during storage at low temperature, and the preconditioning treatment did not affect the total percentage of alcohols either.

Two esters were detected in the cuticular wax of zucchini fruit (Table 1). It was noticeable that 1-monostearin content was predominant over 1-monopalmitin content in both varieties and storage times. The percentage of 1-monostearin decreased in response to low temperature, with a reduction of 13% observed in the cold-tolerant variety, 1.7% in cold-sensitive variety, and 4.4% when ‘Sinatra’ fruit was preconditioned. The percentage of 1-monopalmitin also diminished with cold storage in both varieties.

### Expression Profiles of Cuticle Related Genes During Postharvest

**Figure 4:**
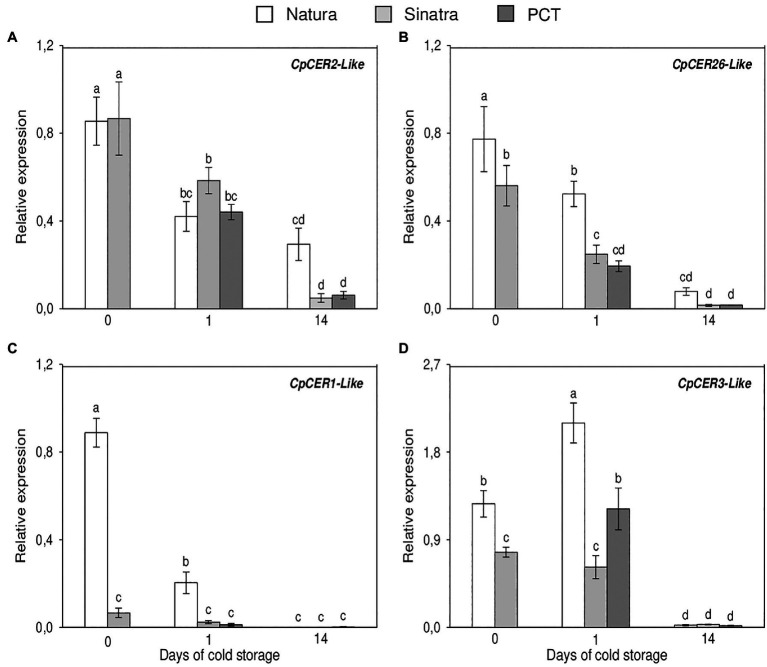
Relative expression of the VLC-alkane biosynthesis genes *CpCER2-like* **(A)**, *CpCER26-like* **(B)**, *CpCER1-like* **(C)**, and *CpCER3-like* **(D)** in the exocarp of ‘Natura’ and ‘Sinatra’, including PCT ‘Sinatra’ fruit, at harvest and after 1 and 14 days of storage at 4°C. Data presented are means ± SE of triplicate samples of six fruits each. Different letters indicate significant differences according to Duncan’s test (*p* < 0.05).

The relative expression of the VLC-alkane biosynthesis genes analyzed is shown in Figure 4. *CpCER2-like* transcripts only exhibited significant differences after 14 days of storage at low temperature in the variety ‘Natura’. By contrast, the levels of *CpCER26-like* mRNA were 38% higher in the cold-tolerant variety with respect to the cold-sensitive one at harvest. Although in both varieties the expression values decreased, these significant differences were maintained and even increased by about 2-fold, after being kept at 4°C for 1 day. Nevertheless, no differences were found at the end of the storage period. With respect to *CpCER1-like* expression, it was about 13- and 8-fold higher in ‘Natura’ as compared with ‘Sinatra’ at harvest and after 24 h of exposure to cold, respectively. These results are in agreement with those found in the RNA-Seq analysis, where *CpCER1-like* was the DEG with the highest differences between freshly-harvested fruit from both varieties. After 14 days at 4°C, the expression values fell in all the fruits to barely detectable levels. Before the storage, the accumulation of the transcripts encoding for CpCER3-like was 64% higher in the cold-tolerant variety ‘Natura’ as compared to the cold-sensitive ‘Sinatra’. These differences were accentuated after 1 day of cold stress, with ‘Natura’ reaching values that were about 3.4-fold higher than ‘Sinatra’. PCT also affected *CpCER3-like* gene expression, with the treated ‘Sinatra’ fruit showing values about 2-fold higher than the control. As with *CpCER1-like*, *CpCER3-like* mRNA sharply decreased after a long-term storage at low temperature.

**Figure 5:**
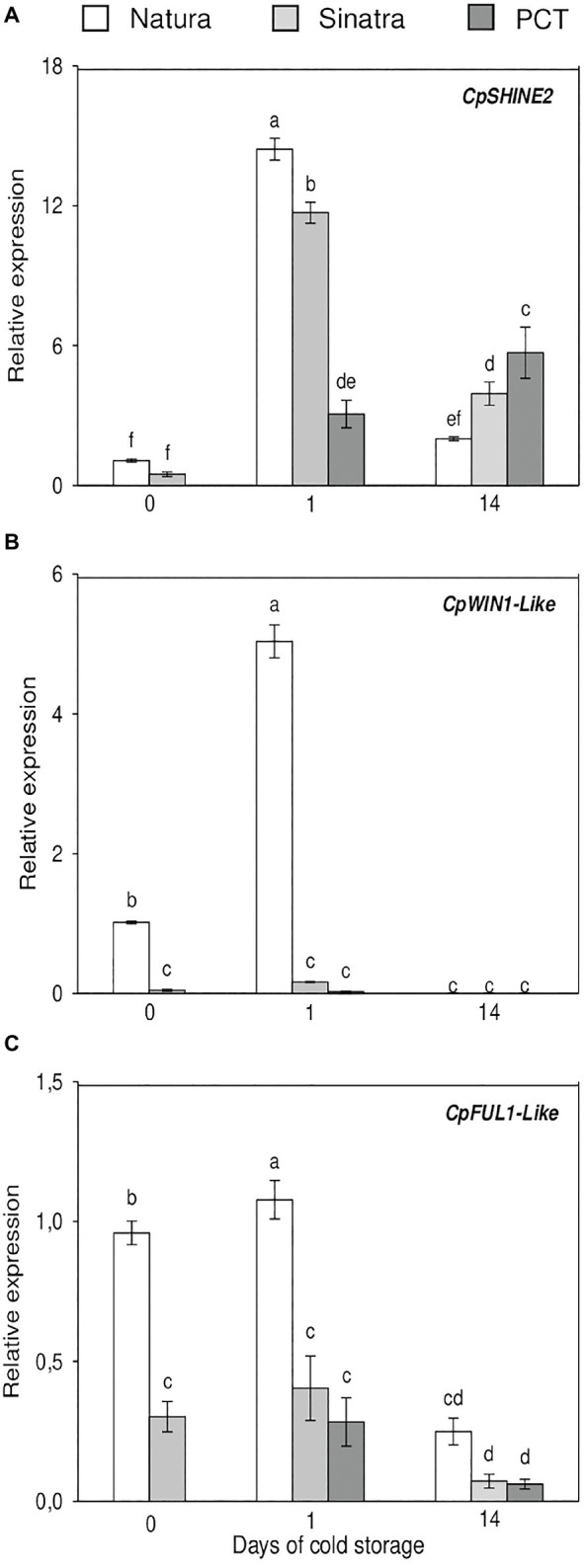
Relative expression of the transcription factors *CpSHINE2* **(A)**, *CpWIN1-like* **(B)**, and *CpFUL1-like* **(C)** in the exocarp of ‘Natura’ and ‘Sinatra’, including PCT ‘Sinatra’ fruit, at harvest and after 1 and 14 days of storage at 4°C. Data presented are means ± SE of triplicate samples of six fruits each. Different letters indicate significant differences according to Duncan’s test (*p* < 0.05).

Expression results of the cuticle-related transcription factors analyzed are represented in Figure 5. *CpSHINE2* gene expression was induced in both varieties when fruits were exposed to 4°C for 1 day, with this increase being significantly higher in the cold-tolerant variety ‘Natura’. After 14 days, the expression levels dropped in ‘Natura’ and ‘Sinatra’ but remained significantly higher in the latter. PCT fruit exhibited the highest accumulation of *CpSHINE2* transcripts at this time. The expression trend of *CpWIN1-like* was the same as *CpSHINE2* in the case of ‘Natura’ fruit; however, the results showed a differential trend between both genes in the cold-sensitive variety ‘Sinatra’. Whereas *CpSHINE2* expression was induced by low temperature, *CpWIN1-like* did not show differences with the postharvest storage, and its values were very low. The high expression values found for this transcription factor in ‘Natura’ exocarp is remarkable, being about 21- and 30-fold higher when compared with ‘Sinatra’. *CpFUL1-like* mRNA accumulation was also greater in the exocarp of ‘Natura’ fruit, about 3- and 2.7-fold, before the postharvest storage and after 24 h of exposure to cold as well, respectively. In this case, the differences found after 14 days at low temperature were not significant.

## Discussion

The implication of the cuticular waxes in the resistance to cold stress during postharvest storage of zucchini was investigated in the present study. A summary of the low temperature effects on the main quality parameters, weight loss, chilling-injury (CI) index, and firmness, throughout storage at 4°C is provided in Supplementary Table S3. These data show that Natura fruit, as well as fruit of Sinatra under a preconditioned treatment, were more tolerant to cold storage that Sinatra fruit, that were more sensitive and with more chilling injuries. Fruits from the more resistant variety ‘Natura’ showed a well-developed cuticle, whereas fruits from the very sensitive ‘Sinatra’ showed a slight stain that was not uniform, indicating a less developed cuticle in fruits that were more prone to developing CI. Cuticular waxes have been suggested to play an important role in fruit quality, postharvest storability, and pathogen susceptibility during postharvest of horticultural crops (Lara Ayala et al., 2014; Chu et al., 2018), being related to fruit postharvest water loss and resistance to environmental and biotic stresses. In zucchini fruit during cold storage after postharvest, the variety ‘Natura’ lost a smaller amount of water than the variety ‘Sinatra’ (Supplementary Table S3), which was also consistent with a better developed cuticle in ‘Natura’. The chlorophyll leaching assay also showed that the cold-tolerant variety had a more reduced cuticular permeability that the cold-sensitive variety, as was also observed in leaves of *Arabidopsis* that overexpressed genes involved in the biosynthesis of VLCFAs (Yang et al., 2021). Transgenic cucumber overexpressing a gene involved in alkane synthesis also had a lower cuticular transpiration and chlorophyll leaching than the wild type (Wang et al., 2015b). Contradictory results have been reported as to the relevance of cuticle thickness in weight loss during storage, but most of the studies published reveal a positive relation between thickness and a decrease in weight loss, for example in pepper (Parsons et al., 2012). In blueberry, the removal of cuticular wax accelerated postharvest water loss and decay, and hence fruit quality (Chu et al., 2018). In zucchini, we conclude that cuticle thickness is related to chilling tolerance and helps to reduce water loss.

From a previous transcriptomic approach contrasting Natura and Sinatra fruits (Carvajal et al., 2018b), important differences among genes related to cuticle have been detected. Among the up-regulated DEGs in ‘Natura’, genes for wax and cutin synthesis were included. Homologous of these genes have been reported to be implicated cuticle development and also in water maintenance, such as GDSL lipase occluded stomatal pore 1 (At2g04570) from *Arabidopsis* (Tang et al., 2020), or the cytochrome P450, with a high identity with *Arabidopsis* CYP86A2, whose *att1* mutants showed a reduction of all cutin monomers and a consequent increase in cuticle permeability (Xiao et al., 2004). On the other hand, the only DEG up-regulated in ‘Sinatra’ with respect to ‘Natura’ at harvest corresponded with a MBOAT, a homolog to At5g55340, which catalyzes the final step in the synthesis of long-chain linear esters. The differences found at the gene expression level are related with the higher development of the cuticle in ‘Natura’, as observed at the microscopic level as well as its lower permeability; it also highlights the importance of the pre-harvest transcriptomic status on the subsequent postharvest behavior during cold storage.

In fruit of the varieties ‘Natura’ and ‘Sinatra’, differences in cuticular wax load were also detected, both at harvest, with a higher amount of waxes per surface area in ‘Natura’ than in ‘Sinatra’, and a sharp increase after 14 days of cold storage in ‘Natura’. In fruits of the preconditioned ‘Sinatra’, a higher amount was also observed after 14 days at 4°C. These results are supported by the SEM microscopy images, where higher wax crystal deposition was detected in ‘Natura’ and in PCT ‘Sinatra’ fruit surfaces. The preconditioned fruit showed images similar to those found in ethylene-treated orange fruit, where the formation of a new wax cover has been described (Cajuste et al., 2010). In that case, ethylene improved the postharvest behavior related to non-chilling peel pitting and increased cuticular wax yields. These results, together with the higher resistance to cold in ‘Natura’ and the great improvement in the postharvest performance of preconditioned ‘Sinatra’ fruit, point to an involvement of cuticular wax on the defense to cold stress conditions and the maintenance of the fruit water status (Supplementary Table S3).

With respect to the components of the cuticular wax, changes in composition and content during postharvest storage have been characterized in fruits such as apple (Chai et al., 2020), blueberry (Chu et al., 2018), and pear (Wang et al., 2021). These studies revealed that triterpenes and alkanes were, in general, the most prominent wax components of fruit cuticles in many species. The predominance of a wax compound has been associated to the taxonomic family of the species (Lara Ayala et al., 2014). We did not detect triterpenes in zucchini cuticular waxes. In fruits of *Cucumis sativus*, the main components were alkanes, aldehydes, and fatty acids (Wang et al., 2015a), similar to the components found in *Cucurbita pepo*. The most abundant alkanes, in our case, were heptacosane (C27), nonacosane (C29), and hentriacontane (C31); with the last two being the most abundant alkanes in fruit cuticles of many species (Wang et al., 2015a; Wu et al., 2018; Romero and Lafuente, 2020). The amount of alkanes increased in the cold-resistant fruit of ‘Natura’ during the storage at 4°C after 14 days, but remained unchanged in sensitive ‘Sinatra’ fruits, also increasing in PCT ‘Sinatra’ fruit, which indicates a mechanism to overcome chilling damages in which cuticular wax synthesis would be involved. The involvement of the alkanes in the defense to stresses has been reported for other species. In a study conducted with 50 pepper accessions, Parsons et al. (2012) found a positive correlation between water loss and alkane content/composition. When analyzing 10 apple cultivars during cold storage, the lowest alkane content was found in the ‘Red Star’ cultivar., which showed the highest weight loss rate during the storage period (Chai et al., 2020). In apple fruit peel, low temperatures induced the alkane-forming pathway and resulted in the accumulation of VLC-alkanes (C22, C27, C29, and C31; Hao et al., 2017), the same accumulation of C27, C29, and C31 alkanes detected in ‘Natura’ fruit and also in preconditioned fruit of ‘Sinatra’, which points to the implication of these compounds in chilling tolerance of zucchini fruit.

We have detected that the content of esters was higher in ‘Natura’ than in ‘Sinatra’ at harvest, whereas the fatty acids content showed the contrary behavior. This could be due to a higher rate of ester hydrolysis in the cold-sensitive variety. In apple fruit, an increase in the content of fatty acids has been described due to the hydrolysis of esters (Veraverbeke et al., 2001). Samuels et al. (2008) have described that most cuticular waxes are generated from the elongation of C16 and C18 free fatty acids, including two well characterized metabolic pathways for the formation of alkanes and for the production of primary alcohols and wax esters. In our analysis, we detected only two esters in zucchini fruit: 1-monopalmitin and 1-monostearin. These two esters were also the only glyceride compounds identified in all 35 pear cultivars studied when the chemical composition of the cuticular wax in mature fruits was analyzed (Wu et al., 2018). The content of 1-monopalmitin and 1-monostearin diminished during the cold storage in both zucchini varieties, observing a greater decrease in ‘Natura’ than ‘Sinatra’ fruit. This behavior is indicative of an induction of the alkane biosynthesis pathway and the inhibition of the ester’s biosynthesis pathway in the cold-tolerant fruit kept at 4°C. This finding is supported by different studies such as that of Wang et al. (2015a), where the knocked-down expression of CsCER1 in transgenic cucumber plants caused a decrease in alkanes content as well as an increase in esters, whereas overexpression of CsCER1 showed the opposite response. In a similar way, transgenic cucumber lines with an abnormal expression of *CsWAX2*, an *Arabidopsis CER3* homolog involved in alkane synthesis, showed an increased amount of esters in cuticular waxes, which decreased with the overexpression of this gene (Wang et al., 2015b). The alternation between the formation of alkanes and wax esters also seems to be regulated by abscisic acid (ABA). In cucumber, the expression of *CsCER1* was shown to be induced by this phytohormone (Wang et al., 2015a), whereas the ABA deficiency orange mutant Pinalate showed an induction in the gene *CsWSD1-like*, responsible for wax ester formation (Romero and Lafuente, 2020). These results suggest that ABA could act as a negative regulator of wax ester formation, and a positive regulator of alkane formation. In a previous work, we described that ‘Natura’ fruit drastically increased the ABA content during the first days of exposure to low temperature, whereas ‘Sinatra’ fruit did not show significant differences in ABA content during storage (Carvajal et al., 2017). This ABA response could be associated with the changes observed in the content of alkanes and esters in the two zucchini varieties, and should be investigated in the future.

It is obvious from this research that cuticular waxes, specifically alkanes, play an important role in defense against low temperature stress in fruits of *Cucurbita pepo*. Fruits of ‘Natura’, a cold resistant variety, and preconditioned ‘Sinatra’ had a higher content of alkanes after cold storage, and this correlated with a lower weight loss and CI damage during postharvest, as well as greater firmness. The biosynthetic pathway of cuticular waxes and regulatory genes have been researched and described in model species and in some fruits, but little is known in zucchini fruit. In this study, we analyzed the expression of 4 genes involved in proper cuticle development, and specifically involved in VLC-alkane biosynthesis. *CpCER2-like* only showed differences in the cold-tolerant variety ‘Natura’ at the end of the storage period. On the contrary, *CpCER26-like* gene expression was significantly higher in ‘Natura’ at harvest and after 1 day of cold exposure. Both genes, CER2 and CER26, are important components of the VLCFA elongation process (Haslam et al., 2017), with the *Arabidopsis cer2* mutant showing an impaired production of wax components longer than 28 carbons, and *cer2*6 mutant affected in the production of wax components longer than 30 carbons (Pascal et al., 2013). The higher expression of both genes in Natura fruit during storage, explains the accumulation of nonacosane (C29) and hentriacontane (C31).

The expression of the zucchini homolog genes, *CpCER1-like* and *CpCER3-like,* has been studied in this work. *CpCER1-like* mRNA was highly accumulated when ‘Natura’ fruit were harvested, whereas in ‘Sinatra’, very low levels were measured. Our results are supported by those found in *Arabidopsis cer1* insertional mutants, which showed a reduction in heptacosane (C27), nonacosane (C29), hentriacontane (C31), and tritriacontane (C33), with its overexpression resulting in an accumulation of these components (Bourdenx et al., 2011). The expression of *CpCER1-like* in zucchini decreased with storage at 4°C, similar to the results found by (Bourdenx et al., 2011), which reported a decreased expression of the CER1 gene in *Arabidopsis* plants subjected to dark and cold treatments. This behavior is in agreement with the trend described in previous works (Wang et al., 2015b) for its homolog *CsWAX2*, which was induced after 24 h of cold stress in cucumber plants. These authors also described a significant reduction in the concentration of the alkanes, pentacosane (C25), heptacosane (C27), nonacosane (C29), and hentriacontane (C31), in fruit from *CsWAX2* RNAi lines, as well as an increase of them in the *CsWAX2* overexpression lines. In zucchini fruit, the large difference found in *CpCER1-like* and *CpCER3-like* gene expression between varieties at harvest time and after 1 day of storage could explain, in part, the accumulation VLC-alkanes during cold stress.

Regarding the possible role of transcriptional regulators in wax biosynthesis, it has been reported that several members of the APETALA2/ETHYLENE-RESPONSIVE ELEMENTBINDING PROTEINS (AP2/EREBP) family regulate cuticle-related genes. In this work, we analyzed the expression of two cuticle-related AP2/EREBP transcription factors, *CpSHINE2* and *CpWIN1-like.* Cold storage induced *CpSHINE2* mRNA accumulation in both varieties in the short-term, with the increase being greater in the transcription detected in ‘Natura’. On the contrary, this induction occurred in the long-term in PCT ‘Sinatra’ fruit. However, *CpWIN1-like* only showed a sharp increase in the cold-tolerant ‘Natura’ fruit after being exposed to low temperature, whereas no changes were detected in ‘Sinatra’. Recently, Zhang et al. (2019) reported that the ectopic expression of the apple gene *MdSHINE2* in *Arabidopsis* resulted in a higher accumulation of wax crystals, and an increase in the alkanes, alcohols, aldehydes, and fatty acids wax components. These results correlated with an induction of CER1, CER3, and WIN1 gene expression, as well as an increase in drought tolerance. *WAX INDUCER1/SHINE1* has been associated with the induction of different wax biosynthesis genes such as *CER1* and *CER2* (Broun et al., 2004). *Arabidopsis* plants overexpressing *WIN1/SHN1* also showed enhanced drought tolerance (Aharoni et al., 2004). These results provide evidence that these genes play an important role on the regulation of the cuticular wax biosynthesis in zucchini fruit subjected to stress due to low temperatures.

Several MADS-box genes have been associated with the ripening process in tomato fruit, such as the FRUITFULL homologues, which may play a role in cuticle formation (Bemer et al., 2012; Shima et al., 2013; Fujisawa et al., 2014). In zucchini fruit, the homolog to tomato TDR4/FUL1, *CpFUL1-like*, was significantly expressed in the cold-tolerant ‘Natura’ at harvest and after a short-term storage at 4°C. This response could be related with the lower percentage of weight loss observed in these fruits according to Bemer et al. (2012), who described an increase in the water loss rate in harvested tomato FUL1/2 RNAi fruits.

This study is the first comprehensive analysis of the cuticular wax structure, load, and composition in *Cucurbita pepo* fruit during postharvest, and its involvement in the defense against chilling injury, proving the importance of the biosynthesis of VLC-alkanes and its transcriptional regulation during the adaptation of the zucchini fruit to low temperatures. Overall, the results obtained can be the basis of future functional studies, and could serve in the identification of markers for the selection of cold-resistant varieties in zucchini breeding programs.

## Data Availability Statement

The original contributions presented in the study are included in the article/Supplementary Material, further inquiries can be directed to the corresponding authors.

## Author Contributions

DG and MJ designed the project and were responsible for funding acquisition. FC and FP conducted the research and analyzed the results. RJ-M and AC-C collaborated in part of the investigation. FC, FP, and DG wrote the original draft with contributions by MJ. All authors have read and accepted the final manuscript.

## Funding

This work was supported by the Ministerio de Ciencia, Innovacion y Universidades (Project AGL2017-82885-C2-2-R and Project PID2020-118080RB-C22). AC-C is being funded by FPI Grants (MEC).

## Conflict of Interest

The authors declare that the investigation was conducted in the absence of any financial interests that could be interpreted as a potential conflict of interest.

## Publisher’s Note

All claims expressed in this article are solely those of the authors and do not necessarily represent those of their affiliated organizations, or those of the publisher, the editors and the reviewers. Any product that may be evaluated in this article, or claim that may be made by its manufacturer, is not guaranteed or endorsed by the publisher.

## Footnotes

^1^ https://primer3.ut.ee/
