# Supplementary material for: Changes in Morphology, Metabolism and Composition of Cuticular Wax in Zucchini Fruit During Postharvest Cold Storage
Source: Front Plant Sci. 2021 Dec 7;12:778745. doi: 10.3389/fpls.2021.778745 (PMC8691734; doi:10.3389/fpls.2021.778745)
Supplement: Supplementary file 1 [file Data_Sheet_1.ZIP › Supplementary_Material/Supplementary_Material_Figure_S1.docx]

**Supplementary Figure S1**

Sinatra

Natura


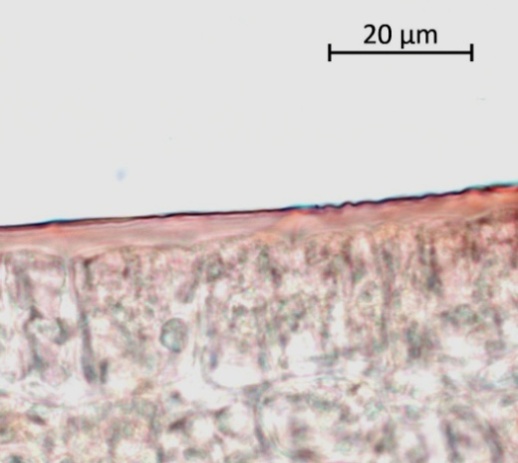

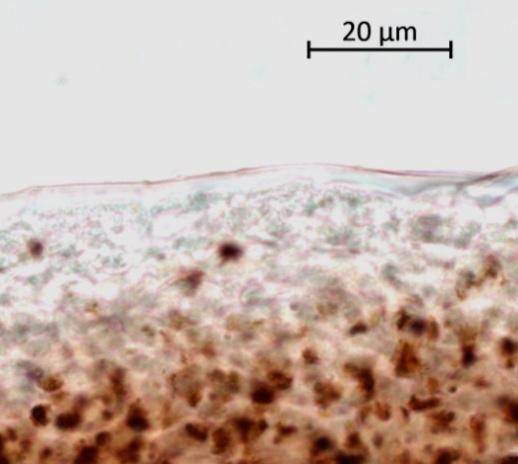


Supplementary Figure S1. Zucchini exocarp cryosections from freshly-harvested fruit the variety ‘Natura’ and ‘Sinatra’ stained with Oil red O and visualized by light microscopy. Scar bars: 20 µm.
