# Supplementary material for: Changes in Morphology, Metabolism and Composition of Cuticular Wax in Zucchini Fruit During Postharvest Cold Storage
Source: Front Plant Sci. 2021 Dec 7;12:778745. doi: 10.3389/fpls.2021.778745 (PMC8691734; doi:10.3389/fpls.2021.778745)
Supplement: Supplementary file 1 [file Data_Sheet_1.ZIP › Supplementary_Material/Supplementary_Material_Figure_S2.docx]

**
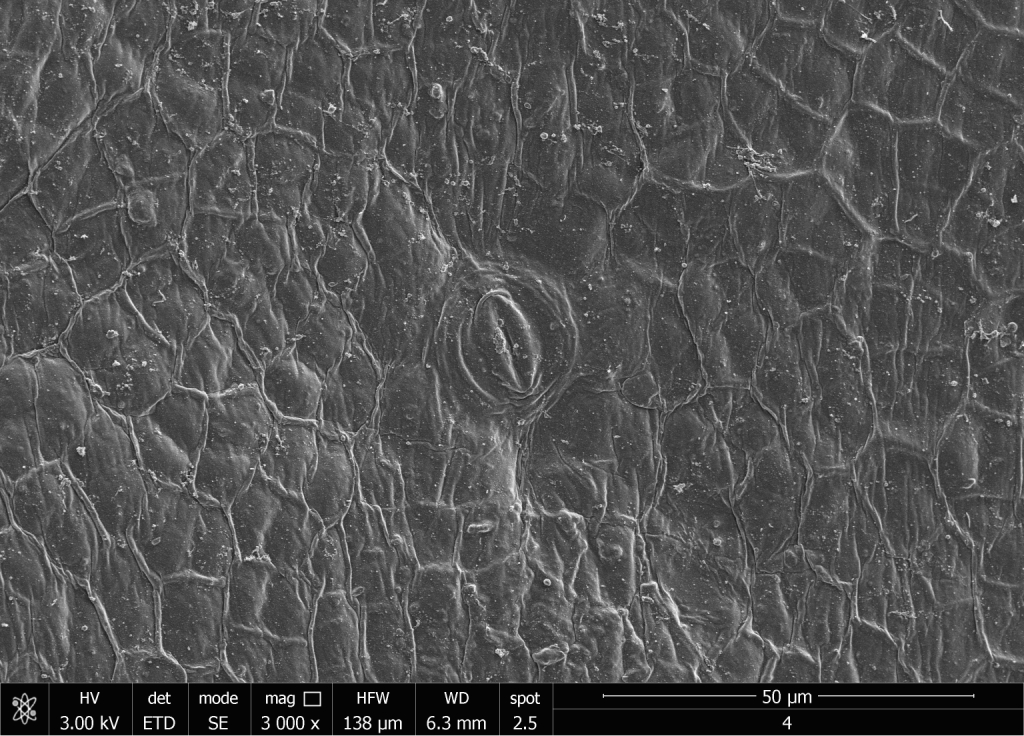
Supplementary Figure S2**


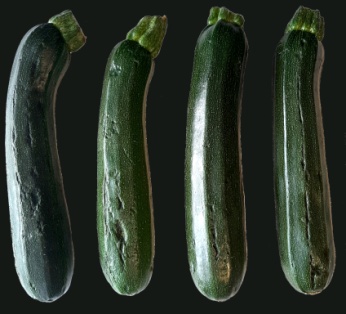


**Sinatra**


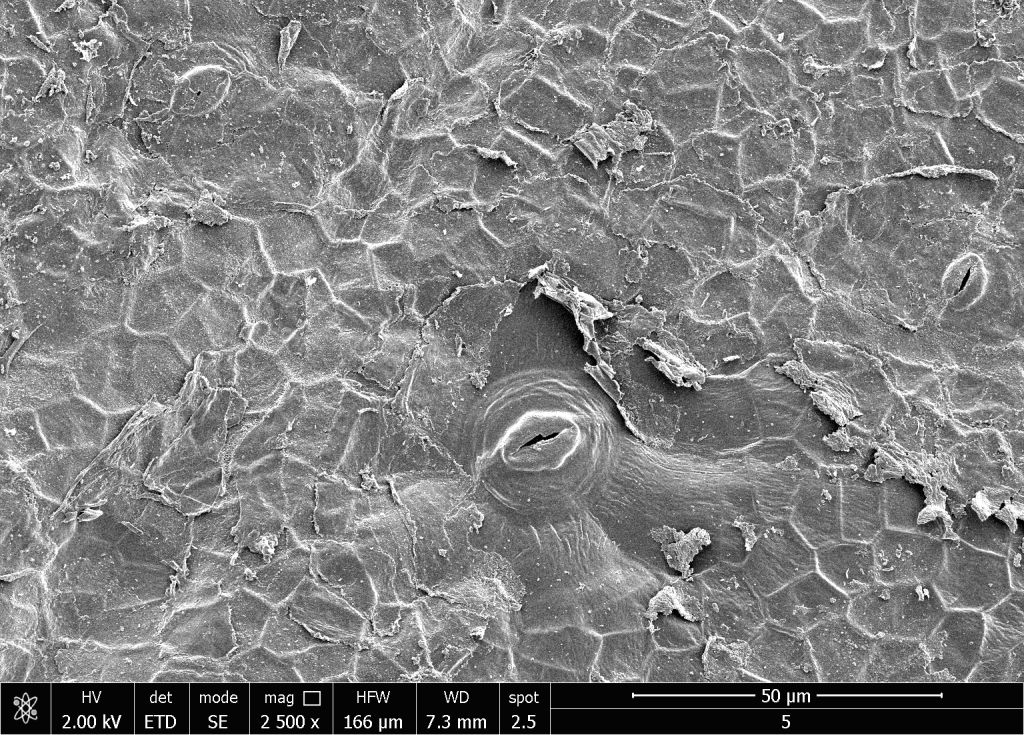


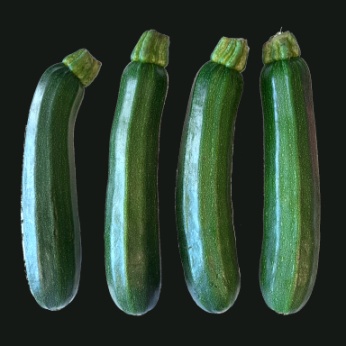


**PCT**

Supplementary Figure S2. Scanning electron micrographs of zucchini exocarp surface from ‘Sinatra’ fruit control and preconditioned (PCT) after 14 days of storage at 4ºC. Scar bars: 50 µm.
