# Supplementary material for: Changes in Morphology, Metabolism and Composition of Cuticular Wax in Zucchini Fruit During Postharvest Cold Storage
Source: Front Plant Sci. 2021 Dec 7;12:778745. doi: 10.3389/fpls.2021.778745 (PMC8691734; doi:10.3389/fpls.2021.778745)
Supplement: Supplementary file 1 [file Data_Sheet_1.ZIP › Supplementary_Material/Supplementary_Material_Table_S1.docx]

# Supplementary Table S1

| **Table S1.** Primers pairs used to perform quantitative RT-PCR | | | | | | |  |
| --- | --- | --- | --- | --- | --- | --- | --- |
|  | **Locus identifier** | **Name** |  | **Primer sequence (5′ to 3′)** | | |  |
|  |  |  |  | **Forward** |  | **Reverse** |  |
|  |  |  |  |  |  |  |  |
|  | Cp4.1LG02g03940 | CpCER2-like |  | CGAGCGAAGAGGTGAGAAAT |  | CGCACGACTGTAAACCAAATC |  |
|  | Cp4.1LG17g02960 | CpCER26-like |  | ATCTATCTCCGCCGTCCTTT |  | GCTTATTTTCTTCCTCCCCTGC |  |
|  | Cp4.1LG04g12660 | CpCER3-like |  | CTTACACTTGCGACCGAGAGA |  | AAGCCAATATGGGAGGGACT |  |
|  | Cp4.1LG17g02820 | CpCER1-like |  | GGATAGTGGGAGATGGATTGG |  | GCTCATCACTCTTCTCGGCA |  |
|  | Cp4.1LG06g03420 | CpSHINE2 |  | TGGCTAGGGACATTGACACAG |  | GCTTTCAGATCGGTCTTTCG |  |
|  | Cp4.1LG04g09380 | CpWIN1-like |  | CGACCCATGACACCAACATTACT |  | GATGGAGTCCGACAGCATTT |  |
|  | Cp4.1LG03g15390 | CpFUL1-like |  | GAATGAGACGGTTTGGGTTG |  | TGGATGTTGTTGGTGCTTGT |  |
|  | Cp4.1LG17g03150 | EF-1α |  | GCTTGGGTGCTCGACAAACT |  | TCCACAGAGCAATGTCAATGG |  |
