# Supplementary material for: Changes in Morphology, Metabolism and Composition of Cuticular Wax in Zucchini Fruit During Postharvest Cold Storage
Source: Front Plant Sci. 2021 Dec 7;12:778745. doi: 10.3389/fpls.2021.778745 (PMC8691734; doi:10.3389/fpls.2021.778745)
Supplement: Supplementary file 1 [file Data_Sheet_1.ZIP › Supplementary_Material/Supplementary_Material_Table_S2.docx]

# Supplementary Table S2

| **Table S2.** Differential expressed genes (DEGs) related to with the cuticle metabolism in freshly-harvested exocarp of ‘Natura’ fruit compared to ‘Sinatra’ and their homologous in *Arabidopsis thaliana* or *Solanum lycopersicum* | | | | | | | | | | | |
| --- | --- | --- | --- | --- | --- | --- | --- | --- | --- | --- | --- |
|  |  |  |  |  |  |  |  |  |  |  |  |
|  | ***Cucurbita pepo*** |  |  |  |  | ***Arabidopsis thaliana or Solanum lycopersicum*** | |  |  |  |  |
|  | **Locus identifier** | **Description** |  | **Fold Change** |  | **Locus identifier** | **Description** | **Function** |  | **References** |  |
|  |  |  |  |  |  |  |  |  |  |  |  |
|  | Cp4.1LG17g02820 | Fatty acid hydroxylase superfamily |  | 3.30 |  | At1g02205 | Very-long-chain aldehyde decarbonylase CER1 | Aldehyde decarbonylase core component of a very-long-chain alkane synthesis complex involved in epicuticular wax biosynthesis |  | Bourdenx et al. (2011), Bernard et al. (2012) |  |
|  | Cp4.1LG18g01010 | GDSL esterase/lipase family |  | 3.13 |  | At5g33370 | GDSL esterase/lipase | Involved in acyltransfer or hydrolase reactions with lipid and non-lipid substrates. Mutants are defective in cuticle formation |  | Hong et al. (2017) |  |
|  | Cp4.1LG13g01490 | Cytochrome P450, putative |  | 2.86 |  | At4g00360 | Cytochrome P450 86A2 | Omega-hydroxylation of fatty acids with chain lengths from C12 to C18 |  | Xiao et al. (2004) |  |
|  | Cp4.1LG01g04680 | 3-ketoacyl-CoA synthase |  | 2.78 |  | At5g04530 | 3-ketoacyl-CoA synthase 19 | Biosynthesis of VLCFA |  | Joubès et al. (2008) |  |
|  | Cp4.1LG04g09380 | Ethylene-responsive transcription factor WIN1 |  | 2.74 |  | At1g15360 | Ethylene-responsive transcription factor WIN1 | Promotes cuticle formation by inducing the expression of enzymes involved in wax biosynthesis |  | Kannangara et al. (2007), Oshima et al. (2013) |  |
|  | Cp4.1LG19g01490 | Non-specific lipid-transfer protein |  | 2.58 |  | At5g01870 | Non-specific lipid-transfer protein 10 | Non-specific lipid-transfer proteins may play a role in wax or cutin deposition in the cell walls of expanding epidermal cells |  | Li-Beisson et al. (2013) |  |
|  | Cp4.1LG02g13950 | MADS box transcription factor |  | 2.32 |  | Solyc06g069430 | FRUITFULL-like MADS-box 1 | Involve in cuticle assembly |  | Berner et al. (2012), Shima et al. (2013) |  |
|  | Cp4.1LG15g08700 | GDSL esterase/lipase |  | 2.26 |  | At2g04570 | GDSL esterase/lipase | Involved in acyltransfer or hydrolase reactions with lipid and non-lipid substrates |  | Tang et al. (2020) |  |
|  | Cp4.1LG00g05770 | Non-specific lipid-transfer protein |  | 2.01 |  | At5g01870 | Non-specific lipid-transfer protein 10 | Non-specific lipid-transfer proteins may play a role in wax or cutin deposition in the cell walls of expanding epidermal cells |  | Li-Beisson et al. (2013) |  |
|  | Cp4.1LG01g13640 | 3-ketoacyl-CoA synthase |  | 1.96 |  | At1g68530 | 3-ketoacyl-CoA synthase 6 | Required for elongation of C24 fatty acids |  | Millar et al. (1999), Fiebig et al. (2000) |  |
|  | Cp4.1LG15g04890 | O-acyltransferase WSD1 |  | 1.83 |  | At5g37300 | O-acyltransferase WSD1-like | Bifunctional wax ester synthase/diacylglycerol acyltransferase involved in cuticular wax biosynthesis |  | Li et al. (2008) |  |
|  | Cp4.1LG04g02550 | GDSL esterase/lipase family |  | 1.69 |  | At2g04570 | GDSL esterase/lipase | Involved in acyltransfer or hydrolase reactions with lipid and non-lipid substrates |  | Tang et al. (2020) |  |
|  | Cp4.1LG03g10200 | 3-ketoacyl-CoA synthase |  | 1.62 |  | At5g43760 | 3-ketoacyl-CoA synthase 20 | Biosynthesis of VLCFAs from 22 to 26 carbons |  | Lee et al. (2009) |  |
|  | Cp4.1LG14g06330 | HXXXD-type acyl-transferase family protein |  | 1.56 |  | At5g23940 | BAHD acyltransferase DCR | Required for incorporation of 9(10),16-dihydroxy-hexadecanoic acid into cutin |  | Panikashvili et al. (2009) |  |
|  | Cp4.1LG02g05730 | 3-ketoacyl-CoA synthase |  | 1.51 |  | At2g26250 | 3-ketoacyl-CoA synthase 10 | Contributes to cuticular wax and suberin biosynthesis |  | Yephremov et al. (1999), Pruitt et al. (2000) |  |
|  |  |  |  |  |  |  |  |  |  |  |  |
|  | Cp4.1LG02g00790 | Membrane bound O-acyl transferase |  | -1.52 |  | At5g55340 | Probable long-chain-alcohol O-fatty-acyltransferase 5 | Catalyzes the final step in the synthesis of long-chain linear esters |  | Chen et al. (2007) |  |

**Supplementary Material References**

Bernard, A., Domergue, F., Pascal, S., Jetter, R., Renne, C., Faure, J.-D., et al. (2012). Reconstitution of Plant Alkane Biosynthesis in Yeast Demonstrates That *Arabidopsis* ECERIFERUM1 and ECERIFERUM3 Are Core Components of a Very-Long-Chain Alkane Synthesis Complex. *Plant Cell* 24, 3106–3118. doi:10.1105/tpc.112.099796.

Bourdenx, B., Bernard, A., Domergue, F., Pascal, S., Léger, A., Roby, D., et al. (2011). Overexpression of Arabidopsis ECERIFERUM1 Promotes Wax Very-Long-Chain Alkane Biosynthesis and Influences Plant Response to Biotic and Abiotic Stresses. *Plant Physiology* 156, 29–45. doi:10.1104/pp.111.172320.

Chen, Q., Steinhauer, L., Hammerlindl, J., Keller, W., & Zou, J. (2007). Biosynthesis of phytosterol esters: identification of a sterol O-acyltransferase in Arabidopsis. *Plant Physiology*, 145(3), 974-984. doi:10.1104/pp.107.106278.

Fiebig, A., Mayfield, J. A., Miley, N. L., Chau, S., Fischer, R. L., & Preuss, D. (2000). Alterations in CER6, a gene identical to CUT1, differentially affect long-chain lipid content on the surface of pollen and stems. *The Plant Cell*, 12(10), 2001-2008. doi:10.1105/tpc.12.10.2001.

Hong, L., Brown, J., Segerson, N.A., Rose, J.K.C, Roeder, A.H.K. (2017). CUTIN SYNTHASE 2 Maintains Progressively Developing Cuticular Ridges in Arabidopsis Sepals. *Molecular Plant* 10 (4), 560–574. doi:10.1016/j.molp.2017.01.002.

Joubès, J., Raffaele, S., Bourdenx, B., Garcia, C., Laroche-Traineau, J., Moreau, P., et al. (2008). The VLCFA elongase gene family in Arabidopsis thaliana: phylogenetic analysis, 3D modelling and expression profiling. *Plant Molecular Biology* 67, 547–566. doi:10.1007/s11103-008-9339-z.

Kannangara, R., Branigan, C., Liu, Y., Penfield, T., Rao, V., Mouille, G., et al. (2007). The transcription factor WIN1/SHN1 regulates cutin biosynthesis in Arabidopsis thaliana. *The Plant Cell*, 19(4), 1278-1294. doi:10.1105/tpc.106.047076.

Lee, S. B., Jung, S. J., Go, Y. S., Kim, H. U., Kim, J. K., Cho, H. J., et al. (2009). Two Arabidopsis 3‐ketoacyl CoA synthase genes, KCS20 and KCS2/DAISY, are functionally redundant in cuticular wax and root suberin biosynthesis, but differentially controlled by osmotic stress. *The Plant Journal*, 60(3), 462-475. doi:10.1111/j.1365-313X.2009.03973.x.

Li, F., Wu, X., Lam, P., Bird, D., Zheng, H., Samuels, L., et al. (2008). Identification of the Wax Ester Synthase/Acyl-Coenzyme A:Diacylglycerol Acyltransferase WSD1 Required for Stem Wax Ester Biosynthesis in Arabidopsis. *Plant Physiology* 148, 97–107. doi:10.1104/pp.108.123471.

Li-Beisson, Y., Shorrosh, B., Beisson, F., Andersson, M. X., Arondel, V., Bates, P. D., et al. (2013). Acyl-lipid metabolism. The Arabidopsis book/American Society of Plant Biologists, 11. doi: 10.1199/tab.0161.

Millar, A. A., Clemens, S., Zachgo, S., Giblin, E. M., Taylor, D. C., & Kunst, L. (1999). CUT1, an Arabidopsis gene required for cuticular wax biosynthesis and pollen fertility, encodes a very-long-chain fatty acid condensing enzyme. *The Plant Cell*, 11(5), 825-838. doi:10.1105/tpc.11.5.825.

Panikashvili, D., Shi, J. X., Schreiber, L., & Aharoni, A. (2009). The Arabidopsis DCR encoding a soluble BAHD acyltransferase is required for cutin polyester formation and seed hydration properties. *Plant Physiology*, 151(4), 1773-1789. doi:10.1104/pp.109.143388.

Pruitt, R. E., Vielle-Calzada, J. P., Ploense, S. E., Grossniklaus, U., & Lolle, S. J. (2000). FIDDLEHEAD, a gene required to suppress epidermal cell interactions in Arabidopsis, encodes a putative lipid biosynthetic enzyme. *Proceedings of the National Academy of Sciences*, 97(3), 1311-1316. doi:10.1073/pnas.97.3.1311.

Oshima, Y., Shikata, M., Koyama, T., Ohtsubo, N., Mitsuda, N., & Ohme-Takagi, M. (2013). MIXTA-like transcription factors and WAX INDUCER1/SHINE1 coordinately regulate cuticle development in Arabidopsis and Torenia fournieri. *The Plant Cell*, 25(5), 1609-1624. doi: 10.1105/tpc.113.110783.

Shima, Y., Kitagawa, M., Fujisawa, M., Nakano, T., Kato, H., Kimbara, J., et al. (2013). Tomato FRUITFULL homologues act in fruit ripening via forming MADS-box transcription factor complexes with RIN. *Plant MolBiol* 82, 427–438. doi:10.1007/s11103-013-0071-y.

Tang, J., Yang, X., Xiao, C., Li, J., Chen, Y., Li, R., et al. (2020). GDSL lipase occluded stomatal pore 1 is required for wax biosynthesis and stomatal cuticular ledge formation. *New Phytologist* 228, 1880–1896. doi:10.1111/nph.16741.

Xiao, F., Goodwin, S. M., Xiao, Y., Sun, Z., Baker, D., Tang, X., et al. (2004). Arabidopsis CYP86A2 represses Pseudomonas syringae type III genes and is required for cuticle development. doi:10.1038/sj.emboj.7600290.

Yephremov, A., Wisman, E., Huijser, P., Huijser, C., Wellesen, K., & Saedler, H. (1999). Characterization of the FIDDLEHEAD gene of Arabidopsis reveals a link between adhesion response and cell differentiation in the epidermis. *The Plant Cell*, 11(11), 2187-2201. doi:10.1105/tpc.11.11.2187.
