# Supplementary material for: Changes in Morphology, Metabolism and Composition of Cuticular Wax in Zucchini Fruit During Postharvest Cold Storage
Source: Front Plant Sci. 2021 Dec 7;12:778745. doi: 10.3389/fpls.2021.778745 (PMC8691734; doi:10.3389/fpls.2021.778745)
Supplement: Supplementary file 1 [file Data_Sheet_1.ZIP › Supplementary_Material/Supplementary_Material_Table_S3.docx]

# Supplementary Table S3

| **Table S3.** Weight loss (%), chilling-injury index, and firmness at central region ‘Natura’ (cold-tolerant variety), ‘Sinatra’ (cold-sensitive variety) and PCT (preconditioned treatment in ‘Sinatra’) zucchini fruit stored at 4 °C | | | | | | | | | |
| --- | --- | --- | --- | --- | --- | --- | --- | --- | --- |
|  |  |  |  |  |  |  |  |  |  |
| **Days of cold storage** |  |  | **Natura** |  | **Sinatra** |  | **PCT** |  | **LSD**  **(*p*<0.05)** |
| 1 | Weight loss (%) |  | 1.25^a^ |  | 1.21^a^ |  | 0.72^b^ |  | 0.22 |
|  | CI-index (0-3) |  | 0.00^a^ |  | 0.00^a^ |  | 0.00^a^ |  | 0.00 |
|  | Firmness (N) |  | 46.41^a^ |  | 42.73^ab^ |  | 38.52^b^ |  | 5.20 |
|  |  |  |  |  |  |  |  |  |  |
| 2 | Weight loss (%) |  | 1.85^a^ |  | 2.06^a^ |  | 1.37^b^ |  | 0.39 |
|  | CI-index (0-3) |  | 0.00^b^ |  | 0.33^a^ |  | 0.00^b^ |  | 0.23 |
|  | Firmness (N) |  | 43.84^a^ |  | 34.40^b^ |  | 42.12^a^ |  | 5.23 |
|  |  |  |  |  |  |  |  |  |  |
| 3 | Weight loss (%) |  | 3.01^a^ |  | 2.50^ab^ |  | 2.37^b^ |  | 0.58 |
|  | CI-index (0-3) |  | 0.23^b^ |  | 0.46^a^ |  | 0.00^c^ |  | 0.21 |
|  | Firmness (N) |  | 44.42^a^ |  | 35.15^b^ |  | 40.71^a^ |  | 4.91 |
|  |  |  |  |  |  |  |  |  |  |
| 5 | Weight loss (%) |  | 4.12^a^ |  | 4.42^a^ |  | 3.72^a^ |  | 0.76 |
|  | CI-index (0-3) |  | 0.78^b^ |  | 1.57^a^ |  | 0.00^c^ |  | 0.59 |
|  | Firmness (N) |  | 42.34^a^ |  | 35.91^b^ |  | 46.45^a^ |  | 5.81 |
|  |  |  |  |  |  |  |  |  |  |
| 10 | Weight loss (%) |  | 6.60^b^ |  | 8.88^a^ |  | 6.03^b^ |  | 1.43 |
|  | CI-index (0-3) |  | 0.86^b^ |  | 1.91^a^ |  | 0.16^c^ |  | 0.60 |
|  | Firmness (N) |  | 39.19^a^ |  | 30.81^b^ |  | 40.67^a^ |  | 6.01 |
|  |  |  |  |  |  |  |  |  |  |
| 14 | Weight loss (%) |  | 7.96^c^ |  | 10.90^a^ |  | 9.18^b^ |  | 0.54 |
|  | CI-index (0-3) |  | 0.92^b^ |  | 2.14^a^ |  | 0.35^b^ |  | 0.59 |
|  | Firmness (N) |  | 40.77^a^ |  | 28.65^b^ |  | 37.18^a^ |  | 7.40 |
|  |  |  |  |  |  |  |  |  |  |
| 14 + 1 day at 20 ºC | Weight loss (%) |  | 9.41^c^ |  | 11.55^a^ |  | 10.31^b^ |  | 0.66 |
|  | CI-index (0-3) |  | 1.00^b^ |  | 2.09^a^ |  | 0.42^c^ |  | 0.43 |
|  | Firmness (N) |  | 38.48^a^ |  | 29.12^b^ |  | 35.28^ab^ |  | 8.33 |
|  |  |  |  |  |  |  |  |  |  |
| Values represent means. Different letters indicate significant differences forthe same storage period according to Duncan’s test (*p*<0.05) | | | | | | | | | |
